# Long-Term Clinical Study of Implants Placed in Maxillary Sinus Floor Augmentation Using Beta-Tricalcium Phosphate

**DOI:** 10.3390/ijerph18199975

**Published:** 2021-09-22

**Authors:** Eugenio Velasco-Ortega, Angela Sierra-Baztan, Alvaro Jiménez-Guerra, Antonio España-López, Iván Ortiz-Garcia, Enrique Núñez-Márquez, Jesús Moreno-Muñoz, José Luis Rondón-Romero, José López-López, Loreto Monsalve-Guil

**Affiliations:** 1 Comprehensive Dentistry for Adults and Gerodontology, Master in Implant Dentistry, Faculty of Dentistry, University of Seville, 41009 Barcelona, Spain; evelasco@us.es (E.V.-O.); sierra.baztan@gmail.com (A.S.-B.); alopajanosas@hotmail.com (A.J.-G.); ajep@us.es (A.E.-L.); ivanortizgarcia1000@hotmail.com (I.O.-G.); enrique_aracena@hotmail.com (E.N.-M.); je5us@hotmail.com (J.M.-M.); jolurr001@hotmail.com (J.L.R.-R.); lomonsalve@hotmail.es (L.M.-G.); 2 Oral Health and Masticatory System Group—IDIBELL (Bellvitge Biomedical Research Institute), University of Barcelona, 08907 Barcelona, Spain

**Keywords:** maxillary sinus floor augmentation, bone substitutes, beta-tricalcium phosphate, bone grafting, sinus lift

## Abstract

Introduction. The aim of this study was to show the long-term clinical outcomes of implants placed in maxillary sinus floor augmentation (MFSA) using beta-tricalcium phosphate (β-TCP). Patients and methods. Maxillary patients were diagnosed for MFSA and used beta- β-TCP. After the lateral sinus surgery, implants were loaded at 6 months with restorations. The clinical follow-up was at 10 years. Results. One hundred and one patients (58 females and 43 males) were treated with MFSA. Twenty-nine patients (28.7%) had a history of periodontitis. Thirty-three patients (32.7%) were smokers. One hundred and twenty-one MFSA, 81 unilateral and 20 bilateral sites, with 234 implants were performed. The average vertical bone height available was 4.92 ± 1.83 mm. The average vertical bone gain obtained was 6.95 ± 2.19 mm following MFSA. The implant cumulative survival rate was 97.2%. Three implants (1.3%) were lost during the healing period. Six implants (2.6%) were lost by peri-implantitis. One hundred and fifteen restorations were placed in the patients. Mean marginal bone loss was 1.93 mm ± 1.03 mm. Six patients (27.3%) showed technical complications. Thirty-six implants (15.3%) in 14 patients (13.9%) were associated with peri-implantitis. Conclusions. This study indicates that treatment with implant-supported restoration by MFSA using β-TCP constitutes a successful implant approach.

## 1. Introduction

Several implant treatments have been proposed to address the implant-prosthetic rehabilitation of the atrophic maxilla including maxillary sinus elevation, the use of short implants, placement of angled implants, zygomatic implants, and pterygoid implants [1,2,3,4]. Maxillary sinus floor augmentation (MSFA) is probably the most predictable and best-performing technique for the creation of sufficient volumetric quantity of bone, through regeneration techniques, necessary to position the implants. Moreover, this surgical implant technique aims to increase bone volume for placement of implants and should also allow the gaining of adequate bone quality for obtaining adequate stability immediately and over a long time [1,2,3,4].

From a clinical perspective, the anatomy of the maxillary sinus can affect the planning of surgical-implant treatment. In such cases, it is necessary to perform an MSFA, using the transalveolar approach [5,6], or through the lateral approach initially described by Tatum 1986 [7]. The choice of the treatment should be primarily based on sinus anatomy (cone beam computer tomography) and health (i.e., absence of sinusitis, cysts), the systemic status of the patient (i.e., smoking), and bone augmentation dimensions [8,9].

The transalveolar approach, when the available bone is between 4 and 6 mm, is less invasive and can shorten the treatment time [10]. Furthermore, the functional loading can be applied relatively earlier because the implant insertion and bone augmentation are performed simultaneously in the same surgery [6,11]. Maxillary sinus elevation by lateral approach and a bone graft is the preferred treatment when the available bone is <4–6 mm. Additionally, when bone height varies between 3 and 5 mm, simultaneous placement of the implants is an optional alternative [12,13]. In MSFA using the lateral window technique, the space created between the maxillary residual ridge and elevated membrane of Schneider is filled with grafting material. MSFA could be implemented as either a pre-implant surgical procedure or be combined with simultaneous implant insertion when the implant can be placed with sufficient primary stability [12,13].

A wide variety of grafting materials have been used in MSFA, including an autogenous bone graft and bone substitutes that are generally categorized as natural transplants (allografts and xenografts) and synthetic materials (alloplastic) [14,15,16]. An autogenous bone graft is generally considered the ideal grafting material because of its osteogenic, osteoinductive, and osteoconductive properties. However, the use of an autogenous bone graft is associated with the risk of donor site morbidity and unpredictable graft resorption. The use of bone substitutes is associated only with osteoconductive properties [1,2,15].

Among the various synthetic products used, one that has demonstrated proven efficacy in bone regeneration [17], alone or in combination, in both animals [18,19,20,21] and humans [22,23,24], is β-TCP. Beta-tricalcium phosphate (β-TCP) is a synthetic bone substitute with a rate of calcium and phosphate agents similar or close to cancellous bone [17]. β-TCP has biocompatible and osteoconductive properties and improves bone regeneration by proliferation and differentiation of osteoblasts and mesenchymal cells to promote bone growth [25,26,27]. Several research studies demonstrated that β-TCP may be used as a bone graft substitute for sinus floor augmentation. Furthermore, β-TCP has been mixed with other graft materials (autogenous bone, allograft, or xenograft) for sinus floor augmentation [28,29,30,31]. These clinical, radiographic, and histological findings provided supporting evidence that β-TCP grafting is gradually replaced by new bone over time and suggest that β-TCP is a good synthetic bone substitute with very similar progress to an autogenous graft [29,30,31].

The placement of implants using the maxillary sinus lift technique, which allows for achievement of the necessary height for insertion, is a safe, predictable, and long-term technique. That is why the objective of this clinical study was to evaluate the long-term clinical results of implant treatments with lateral maxillary sinus elevation in patients.

## 2. Materials and Methods

This clinical study included totally and partially edentulous maxillary patients that presented for treatment in the clinic of Master of Implant Dentistry at the School of Dentistry of Seville, Spain, from January 2009 to December 2013. The study was conducted according to the principles outlined in the Declaration of Helsinki on clinical research involving humans. The ethical committee of the University of Seville approved the study and informed written consent for implant placement was obtained from all patients.

**Figure 1:**
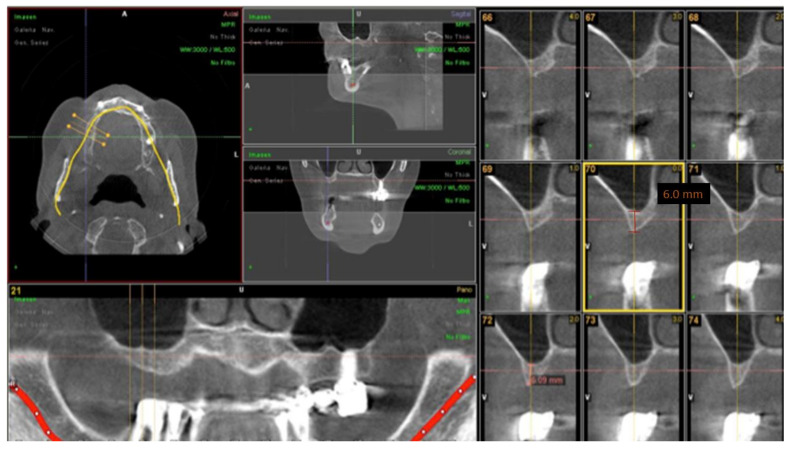
CBCT prior to maxillary sinus elevation surgery.

The inclusion criteria were the need for maxillary sinus elevation in the treatment of patients with implant-supported restorations. The following exclusion criteria were the presence of chronic systemic disease, smoking ≥10 cigarettes/day, bruxism, uncontrolled diabetes or periodontal disease, coagulation disorders, and alcohol or drug abuse. Treatment planning included oral examination, cone beam computerized tomography (CBCT) (Picasso Master 3D^®^, Vatech, Gyeonggi-do, South Korea) (Figure 1), diagnostic casts for intermaxillary relations, and clinical photographs. Patients were informed of all steps of treatment and accepted the clinical protocol of sinus elevation surgery with implant-supported prostheses.

Prior to surgery, the patients received preventive antibiotic therapy (500 mg amoxicillin and 125 mg clavulanic acid 1 h before surgery); they also continued to take the antibiotic postoperatively, 3 capsules daily for 7 days. After surgery, a chlorhexidine mouthwash was prescribed for twice-daily use for 30 days. Ibuprofen (600 mg, 4 times daily) was prescribed for 7 days. All patients were treated under local anesthesia using articaine with adrenaline.

**Figure 2:**
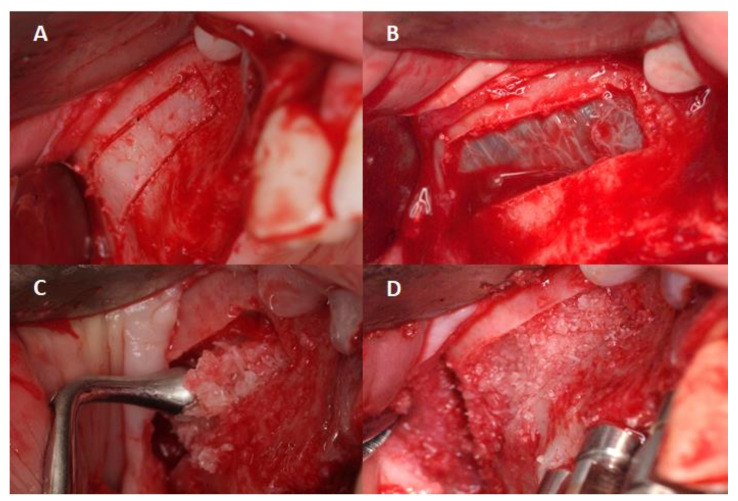
(**A**) Design of the osteotomy using a Piezomed insert of the brand W&H, B1 (REF 05530100); (**B**) design of the lateral window and visualization of the Schneider membrane; (**C**,**D**) once the Schneider membrane has been detached and raised, the cavity is filled with particles of β-TCP.

**Figure 3:**
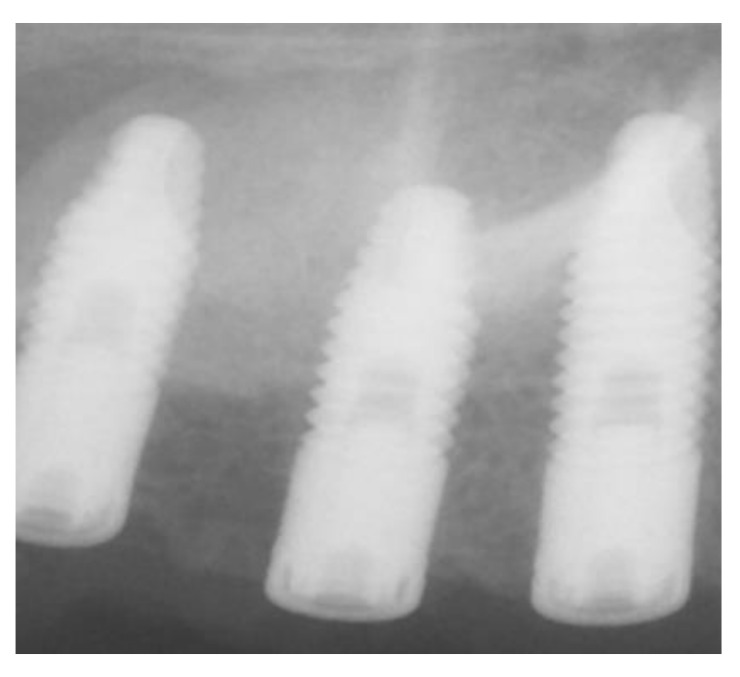
X-ray after installing the implants.

The surgical procedure was performed under local anesthesia. Antisepsis was performed with a one-minute rinse and 5-second friction in the operative area with a gauze soaked in 0.12% chlorhexidine digluconate solution (Lacer Clorhexidina^®^, Lacer, Barcelona, Spain). They were anesthetized locally (Articaina 4% 1:200,000 epinephrine, Ultracain^®^, Normon, Barcelona, Spain). Subsequently, a supracrestal incision was made, using a cold scalpel, with two discharges (mesial and distal); the vestibular flap was then peeled off at full thickness, exposing the maxillary cortex in relation to the maxillary sinus. An osteotomy was performed to create a window access to the maxillary sinus using a Piezomed insert of the brand W&H, B1 (REF 05530100), mounted on a handpiece of piezo surgery (Piezomed^®^, Madrid, Spain), under a 0.9% saline irrigation solution and physiological freezing 2 to 3 mm above the floor. After, a lateral window by osteotomy was performed with a piezoelectric instrument (NSK VarioSurg™, Tokyo, Japan) (Figure 2A–D). The Schneider’s membrane was carefully lifted through proper curettes beginning at the lower edge of the antrostomy and elevating the membrane of the medial and lower walls to allow implant insertion. Following the Schneider’s membrane elevation, the β-TCP (Osteoblast ™, Sarria, Spain) was placed into the maxillary sinus through the lateral window opened from the maxillary sinus wall and the implants were installed from the alveolar crest (Figure 2D and Figure 3).

The radiological preoperative bone height (CBCT) below the maxillary sinus conditioned the implant placement timing. A simultaneous placement was conducted to achieve adequate primary stability of the implant inserted, in cases where the residual bone height was ≥5 mm. If the initial vertical dimension was <5 mm, a delayed placement of implants was carried out.

**Figure 4:**
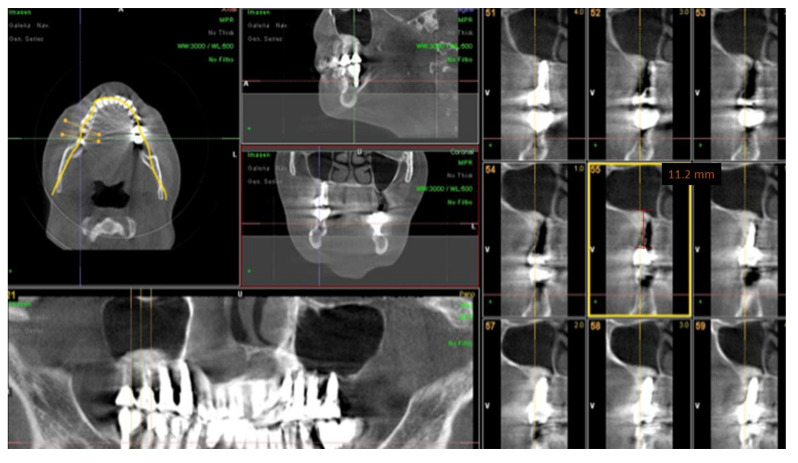
CBCT after maxillary sinus elevation surgery and prosthodontic rehabilitation.

Surgimplant^®^ screw implants (Galimplant^®^, Sarria, Spain) with sandblasted and acid-etched surfaces were used for all implant placements. Insertion torque and resonance frequency analysis were used as methods for measuring implant stability after placement. Since all implants were placed using the implant motor, a standard insertion torque of ≥35 Ncm was set at placement. Finally, resonance frequency analysis was used to confirm the stability of each implant. The stability of the fixture was considered acceptable with an implant stability quotient ranging from 55 to 85. All implants were placed submerged. A new CBCT was performed at six months after the insertion of implants before functional loading for assessment of vertical bone gain was obtained (Figure 4).

After a six-month healing period of insertion of implants, delayed loading was performed when a torque of prosthetic abutments of ≥30 Ncm and ≥55 ISQ value of implants occurred. Implant fixed and removable restorations were manufactured and placed onto the osseointegrated implants.

The criteria used for assessment of survival were implant stability and the absence of radiolucency around the implants, mucosal suppuration, and pain. Follow-up visits were scheduled at 3 and 6 months after implant placement and each year after the first year. In these revisions, the patients were subjected to clinical and radiologic revision of the implants and restorations. Marginal bone loss was evaluated based on digital periapical radiographs taken perpendicular to the long axis of the implants, comparing the difference between the 1-year follow-up radiography and the 10-year follow-up radiography. The analyzed records included patient information (gender, age, dental health, systemic diseases, smoking habits), details about the placed implants (type, number, position, diameter, and length), and the prosthetic restoration including the dates of delivery. Moreover, the analyzed data included all information about any implant failure or biological and technical complication that occurred during the intervention, after the surgery and functional loading, and at each follow-up visit.

All available data from all patients were included in the analyses using the SPSS (SPSS 11.5.0, SPSS, Chicago, IL, USA) package. Descriptive statistics of variables were used to report the general results of the study. For all qualitative variables, values were expressed in absolute terms, and percentages (%) were calculated using the chi-square test. For quantitative variables, the means, standard deviations (SD), medians, ranges, and 95% confidence intervals (CI) were calculated. The similarities in the groups were confirmed by analysis of variance (ANOVA). The Mann–Whitney U non-parametric test was used to compare differences between groups created based on the different risk factors measured. A *p*-value < 0.05 was considered statistically significant.

## 3. Results

One hundred and ninety-eight implants were placed in 101 totally and partially edentulous maxilla patients (treated consecutively), 58 females and 43 males, ranging in age from 28 to 81 years (mean age 56.9). No significant statistical differences were found related to sex and age (Mann–Whitney U test, *p =* 0.1964). Twenty-nine patients (28.7%) had a previous history of periodontitis, 19 females and 10 males; these differences were not statistically significant (chi-square test, *p =* 0.11168). Thirty-three patients (32.7%) were smokers, 17 males and 16 females; these differences were not statistically significant (chi-square test, *p =* 0.20553) Thirty-nine patients (38.6%) exhibited medical conditions (i.e., cardiovascular diseases, diabetes) (Table 1).

Among the included 101 patients, 121 maxillary sinus lift surgeries, 81 unilateral and 20 bilateral, were performed using the surgical protocol of MFSA. The average vertical bone height available prior to the sinus surgery was 4.92 ± 1.83 mm, and the average vertical bone gain obtained was 6.95 ± 2.19 mm following MFSA with β-TCP. These results did not show statistically significant differences between the available bone and acquired bone gain with the variables of patients (age, gender, periodontal background, smoking, and medical conditions) (ANOVA, text -ANalysis Of VAriance-; *p*-value > 0.01). The most common surgical complication found in MFSA patients was the perforation of the membrane of Schneider in 13 patients (21.8%). The perforation is most frequent in younger patients <49 years (10 cases). These differences were statistically significant (chi-square test, *p =* 0.00008) (Table 2).

Two hundred and thirty-four implants were placed in the patients, 120 implants with an external connection and 114 implants with an internal connection. The average follow-up period was 124.05 ± 14.41 months (range: 104–146 months). Nine implants (3.8%) were lost during the clinical follow-up. The cumulative survival rate for all implants was 97.2%. One hundred and thirty-nine implants (59.4%) were placed through a simultaneous surgical approach in patients with a residual bone height ≥ 5 mm (mean ISQ of 64; range 50–76 in vertical and 63; range 49–81, horizontal), and 95 implants (40.6%) with a delayed surgical approach with a residual bone height < 5 mm (mean ISQ of 63; range 48–74 in vertical and 66; range 55–81, horizontal). Seven implants (3%) of delayed placed implants were lost and three implants (0.8%) of simultaneously inserted implants. These differences were statistically significant (Mann–Whitney U non-parametric, *p =* 0.0309).

Seven implants (3%) were 8 mm in length, 95 (40.6%) were 10 mm, and 132 (56.4%) were 12 mm. Thirty-one implants (13.3%) had a diameter of 3.5 mm, 162 (69.2%%) of 4 mm, and 41 (17.5%) had a diameter of 5 mm. Nine implants (2.8%) in eight patients (3.2%) were lost during the follow-up (Table 3). Three implants (1.3%) were lost during the healing period before loading due to a lack of osseointegration. Six implants (2.6%) of the 231 remaining implants were lost by peri-implantitis (Table 4)

During the follow-up period, 36 implants (15.6%) of the remaining implants were associated with peri-implantitis. Peri-implantitis was more frequent, significantly, in smoking patients (27.3%) compared with non-smoking patients (7.4%) (chi-square test, *p* = 0.00658).

The mean marginal bone loss (MBL) was 1.93 mm (SD 1.03 mm), ranging from 1.2 to 5 mm during the follow-up evaluation. In patients with smoking habits, the marginal bone loss was 2.15 ± 0.87 for smoking patients and 1.82 ± 1.09 for non-smoking patients, with no statistical differences (ANOVA; *p =* 0.1367). In patients with medical conditions, the marginal bone loss was 2.61 ± 0.92 compared with 1.86 ± 1.02 for healthy patients, with statistical differences (ANOVA; *p =* 0.0385) (Table 5).

Regarding the prostheses designed, a total of 115 restorations, 37 single-crowns, 73 partially fixed bridges, 4 full-arch fixed restorations, and 1 overdenture, were placed in the patients over the 231 remaining implants after the healing period. Six patients (5.9%) showed some kind of mechanical prosthodontic complications, or loss/fracture of the prosthetic screw (Table 5).

## 4. Discussion

This study evaluated the clinical outcomes in the treatment with MSFA of partially and totally edentulous patients with different prosthodontic restorations with delayed loading of implants. This clinical research demonstrated that, after 10 years of lateral MSFA with β-TCP, the overall cumulative survival rates of implants placed by MSFA procedures were 97.2%.

MSFA can be performed when the residual vertical alveolar bone height is ≤5 mm. Scientific evidence showed that MSFA predictably leads to high implant survival rates, limited peri-implant marginal bone loss, and resulted in few overall surgical and prosthetic complications in maxillary patients [3,4,5,6,7,8]. The lateral osteotomy technique to approach the MFSA for increasing bone volume in the posterior maxilla is a well-established and documented surgical procedure allowing for implant placement [3,13,14,31]. Irrespective of the grafting materials applied (i.e., autogenous bone, allografts, xenografts, alloplastic), MFSA is accompanied by increased implant stabilization and new bone formation [13,14,15].

The findings obtained from the present study observed that β-TCP bone substitute achieved clinical results with substantial vertical bone gain in treated patients where the biomaterial was placed along with delayed and simultaneous implant placement. This study demonstrated that the β-TCP bone substitute achieved an average vertical bone gain of 6.95 ± 2.19 mm for the MFSA. These good clinical observations are confirmed by several studies that reported β-TCP as a safe, osteoconductive, and predictable material to be used in MFSA, alone or combined with other types of bone grafts [24,25,27]. A recent cross-sectional study evaluates the vertical bone gain achieved after the lateral MFSA with β-TCP [16]. One hundred and twenty-eight sinus lift procedures (utilizing a synthetic ceramic containing 99.9% tricalcium phosphate as a bone substitute) and simultaneous implant placements were performed on 119 patients. The implants were evaluated using CBCT at 6 months following placement. Two hundred and sixty implants were placed in the study participants. After six months, in the clinical and radiographic review prior to prosthetic rehabilitation, it was observed that all the surgical sites demonstrated uneventful healing and the implants did not exhibit clinical mechanical looseness, peri-implantitis, or fracture during the follow-up period. The average vertical bone gain obtained was 8.5 ± 0.3 mm [27].

After the MFSA surgery, the presence of adequate clot stability, with the slow resorption of bone substitute materials, and at the same time, the neovascularization and formation of new bone are crucial for the induction of bone growth, by osteoprogenitor cells, in the space between the elevated sinus membrane and the residual bone, independently of the used biomaterial [32,33,34]. The progressive resorption of β-TCP and new bone formation has been reported in several studies [17,25,26]. A 4–5-year follow-up study in MFSA treatment of twenty consecutive patients showed the percentage of β-TCP reabsorption compared with autogenous grafts [35]. Both β -TCP and mandibular bone grafts resulted in a radiographic reduction of the vertical height over the 5 years following MFSA. After an initial height reduction in the first 1.5 years, subsequent changes were minimal. No significant differences were observed between the two types of grafting material [35]. A study compared the changes in bone volume after MFSA using autogenous bone, autogenous bone associated with β-TCP, and β-TCP alone as grafting material, by means of CBCT [28]. Bone volume was obtained in the immediate postoperative period (5–7 days) and at 6 months postoperative in each group. The results showed average resorption of 45.7 ± 18.6% for the autogenous bone group, 43.8 ± 18.4% for the autogenous bone+β-TCP group, and 38.3 ± 16.6% for the β-TCP group. All bone substitute materials tested in the study presented satisfactory results for MFSA procedures regarding the maintenance of graft volume during the healing phase before the insertion of implants [28]. The use of preoperative and postoperative CBCT has gained popularity, and assessment by CBCT for volumetric changes in MFSA using β-TCP has also been reported. The results of a study showed that graft volume decreased over time, both at 6 months after surgery and even at 2.5 years after surgery until 54.9%. Functional loading must be applied to the implant only 6 months after surgery [36].

Implant placement timing (delayed versus simultaneous approach) can be a predictor of implant failures. The residual bone height available is established for the planning and evolution of treatment through the radiological diagnosis [37,38]. Several studies have shown that residual bone height can play an important role in the survival of implants placed with the MSFA surgery [37,38]. In the present study, a simultaneous approach was adopted when the residual height of posterior maxillary bone was ≥5 mm, in order to achieve proper primary stability of the implant inserted. If the residual bone height was <5 mm, a delayed procedure was performed. The implant survival rate obtained in the delayed approach group was 97% while in the simultaneous approach group, it was 99.2%. The findings obtained in the present study were in concordance with other authors, suggesting that a residual bone height < 4–5 mm affects the survival rate of implants placed in MFSA [37,38]. A long-term retrospective study reported the survival of implants placed simultaneously with lateral MSFA during a period of 6–20 years [37]. The overall 10-year and 20-year cumulative survival rates were 95% and 85%, respectively. Cumulative survival rate was significantly higher for implants with ≥3 mm of residual bone height (*n* = 260, 92.4%) than those with <3 mm (*n* = 353, 78.8%) [37]. The results of a systemic review showed a positive relationship between the initial alveolar bone height and implant survival rate with the lateral window technique of MFSA. A meta-regression analysis showed an increasing significant trend of implant survival rate with greater initial bone height for the lateral window technique [38]. Conversely, a clinical study that recommends a simultaneous implant placement when the residual height bone was greater than 4 mm, or a delayed procedure when the residual bone was less than 4 mm, reported a similar implant survival rate in the simultaneous group of patients (98%) and in the delayed group of patients (98.4%), with no statistical difference between the two different surgical approaches [8].

In the present MFSA with β-TCP study, the mean MBL was 1.93 mm ± 1.03 mm during the 10-year follow-up evaluation. In patients with medical conditions, the marginal bone loss was 2.61 ± 0.92 compared with 1.86 ± 1.02 for healthy patients, with statistical differences (ANOVA; *p =* 0.0385). The MBL after MSFA treatment with autogenous bone grafts, or/and bone substitutes, showed a significant gradual peri-implant marginal bone loss was observed from baseline to the 5-year follow-up examination and varied between 0.34 and 2.6 mm [3,4]. A 10-year longitudinal MFSA study reported a cumulative survival rate of implants of 86%, with 80% xenograft bovine bone and 20% autogenous bone [39]. The mean MBL was 1.6 mm±1.0 mm. In clinical studies with a 5-year and 10-year follow-up, MBL after MSFA reported acceptable limits, independently of the bone substitute used and mainly occurred during the early healing phase [3,4,40].

Despite the high survival rate for implants placed in the present study, a global rate of surgical, biologic, and prosthetic complications has also been reported. The perforation of the sinus membrane was the most frequent intraoperative complication (21.8%). This surgical complication is shown in the majority of MFSA clinical studies [3,4,5,27,31]. A recent systematic review included a total of 1162 patients who underwent 1598 lateral access MSFA procedures suffering a mean perforation rate of 30.6% (489 perforations) [41]. Membrane perforation has some clinical implications such as increased susceptibility to infections or an inadequately contained graft and may influence the implant survival rate [40]. The influence of Schneiderian membrane perforations on the survival rate of implants was also a widely and controversially discussed topic [5]. However, several studies and systemic reviews showed no negative effect on the survival rate of implants placed in MFSA patients [4,31,37,42,43].

During the follow-up period of the present study, 36 implants (15.6%) of 231 remaining implants were associated with peri-implantitis. Peri-implantitis was more frequent, significantly, in smoking patients (27.3%) compared with non-smoking patients (7.4%) (chi-square test, *p =* 0.00658). Six implants (2.6%) were lost by peri-implantitis. Previous clinical research has focused on the occurrence of peri-implant diseases in MFSA patients [44,45]. A clinical study reported findings of single implants placed 6 months after MFSA in 53 patients. Fixed restorations were delivered 12 weeks later and reviewed 12 months after function. Mucositis was diagnosed in at least 62% of patients. No peri-implantitis was diagnosed because the study was very short, and peri-implantitis is a longtime-dependent disease [44]. A recent study with a follow-up varying from 1 to 18 years, included 156 patients with 315 implants inserted into MFSA. Seven implants in seven patients were lost for peri-implantitis prior to the beginning of the study (2.2% and 4.5% at implant- and patient-level, respectively). At the implant-level examination, 34 implants presented mucositis (10.8%), and 24 implants exhibited peri-implantitis (7.6%). Implants diagnosed with peri-implantitis had a mean function time of 81.3 ± 27.1 months [42]. Several risk factors of peri-implantitis have been described, including a prior history of periodontitis and smoking [45,46]. Furthermore, smoking is an important risk factor for implant survival rate in MFSA patients, with a significant difference in the long-term cumulative survival rate between the smoking group and non-smoking group [37,45].

Regarding the prostheses designed, a total of 115 restorations, 37 single-crowns, 73 partially fixed bridges, 4 full-arch fixed restorations, and 1 overdenture were placed in the patients. The great majority of patients (94.1%) presented with complication-free implant-supported restorations. Only 6 patients (5.9%) showed some kind of mechanical prosthodontic complications, or loss/fracture of the prosthetic screw. These clinical findings are consistent with previous reports that confirmed minor technical complications occurred in a small number of MFSA patients and included ceramic veneer fractures and screw loosening [3,43].

The main limitation of this clinical study is that it is not a randomized clinical trial, and that TCP is not compared with another biomaterial; the results compare with the literature in this regard. Therefore, the data should be taken with caution, since it is long term.

## 5. Conclusions

This follow-up study, and taking into account its limitations, confirms that the treatment with implants in the posterior maxillary sectors, by means of the maxillary sinus elevation technique, with lateral window and implant placement, in one phase or delayed, is a successful technique long term. The loss of implants, peri-implantitis, the effect of tobacco and systemic diseases, as well as prosthetic problems, behave in a similar way to other implant treatments without sinus elevation.

## Figures and Tables

**Table 1:** Description of patient characteristics.

Patient’s Characteristics			N		N		N		Heading		
			≤49 y		50–64 y		≥65 y				
			28 (27.7%)		42 (41.6%)		31 (30.7%)				
		N/%	*F*	*M*	*F*	*M*	*F*	*M*	N	N	
									*F*	*M*	N
Sex			17	11	24	18	17	14	58 (57.4%)	43 (42.6%)	101
History of periodontitis *	Yes	29 (28.7%)	7	3	6	5	6	2	19	10	101
	No	72 (71.3%)	10	8	18	13	11	12	39	33	
Smokers *	Yes	33 (32.7%)	6	4	5	10	4	3	16	17	101
	No	68 (67.3%)	10	10	20	8	12	8	42	26	
Medical conditions *	Yes	39 (38.6%)	6	6	11	3	5	8	22	17	101
	No	62 (61.4%)	10	8	14	10	10	8	36	26	

(*) = No statistical significance; N = patients; *F* = females; *M* = males; y = years.

**Table 2:** Data regarding marginal bone loss (mm).

	Total				
Age		≤49 years	50–64 years	≥65 years	
	1.93 ± 1.03	2.01 ± 0.99	2.04 ± 1.01	1.69 ± 1.10	
Gender		Female	Male		
	1.93 ± 1.03	1.75 ± 0.94	2.06 ± 1.08		
Periodontal Disease Background		Yes	No		
	1.93 ± 1.03	1.84 ± 1.11	1.95 ± 1.02		
Tobacco		Smokers	Non-Smokers		
	1.93 ± 1.03	2.15 ± 0.87	1.82 ± 1.09		
Type of Connection		External	Internal		
	1.93 ± 1.03	1.76 ± 1.08	2.10 ± 0.96		
Medical History		Yes	No		
	1.93 ± 1.03	2.61 ± 0.92	1.86 ± 1.02		
Follow-up		≤120 months	≥120 months		
	1.93 ± 1.03	1.76 ± 1.12	2.08 ± 0.92		
Prothesis		Crowns	Fixed Prothesis	Rehabilitation	Overdenture
	1.93 ± 1.03	1.53 ± 0.95	2.05 ± 0.98	3 ± 1.63	1.5 ± 1.58

**Table 3:** Description of implant characteristics.

	n	%
Connection type		
External	120	51.3
Internal	114	48.7
Implant diameter (mm)		
3.5	31	13.3
4	162	69.2
5	41	17.5
Implant length (mm)		
8	7	3
10	95	40.6
12	132	56.4
Implant loss	9	2.8

n = implant.

**Table 4:** Distribution of patients according to implant loss.

	Total				
Age		≤49 years	50–64 years	≥65 years	
	8 (13.2%)	4 (14.3%)	2 (4.8%)	2 (6.4%)	
Gender		Female	Male		
	8 (13.2%)	0 (0%)	8 (13.2%)		
Periodontal Disease Background		Yes	No		
	8 (13.2%)	2 (6.9%)	6 (8.3%)		
Tobacco		Smokers	Non-Smokers		
	8 (13.2%)	3 (9.1%)	5 (7.3%)		
Connection		External	Internal		
	8 (13.2%)	5 (9.8%)	3 (6%)		
Medical History		Yes	No		
	8 (13.2%)	3 (12.8%)	5 (8.1%)		
Follow-up		≤120 months	≥120 months		
	8 (13.2%)	3 (6.2%)	5 (9.4%)		
Prothesis		Crowns	Fixed Prothesis	Rehabilitation	Overdenture
	8 (13.2%)	2 (6.7%)	3 (4.5%)	3 (7.5%)	0 (0%)

**Table 5:** Peri-implantitis distribution.

	Total				
Age		≤49 years	50–64 years	≥65 years	
	14 (13.9%)	5 (17.8%)	5 (11.9%)	4 (12.9%)	
Gender		Female	Male		
	14 (13.9%)	4 (9.3%)	10 (17.2%		
Periodontal Disease Background		Yes	No		
	14 (13.9%)	4 (13.7%)	10 (13.9%)		
Tobacco		Smokers	Non-Smokers		
	14 (13.9%)	9 (27.3%)	5 (7.4%)		
Connection		External	Internal		
	14 (13.9%)				
Medical History		Yes	No		
	14 (13.9%)	1 (2.6%)	13 (20.9%)		
Follow-up		≤120 months	≥120 months		
	14 (13.9%)	7 (14.6%)	7 (13.2%)		
Prothesis		Crowns	Fixed Prothesis	Rehabilitation	Overdenture
	14 (13.9%)	3 (10%)	9 (13.6%)	2 (50%)	0 (0%)

## Data Availability

The associated data of a statistical nature, can be requested to: jl.lopez@ub.edu & evelasco@us.es.

## References

[1] Al-Dajani M. (2017). Recent trends in sinus lift surgery and their clinical implications. Clin. Implant. Dent. Relat. Res..

[2] Danesh-Sani S.-A., Loomer P.-M., Wallace S.-S. (2016). A comprehensive clinical review of maxillary sinus floor elevation: Anatomy, techniques, biomaterials and complications. Br. J. Oral. Maxillofac. Surg..

[3] Raghoebar G.-M., Onclin P., Boven G.-C., Vissink A., Meijer H.-J.-A. (2019). Long-term effectiveness of maxillary sinus floor augmentation: A systematic review and meta-analysis. J. Clin. Periodontol..

[4] Starch-Jensen T., Aludden H., Hallman M., Dahlin C., Christensen A.E., Mordenfeld A. (2018). A systematic review and meta- analysis of long-term studies (five or more years) assessing maxillary sinus floor augmentation. Int. J. Oral. Maxillofac. Surg..

[5] Tan W.-C., Lang N.-P., Zwahlen M., Pjetursson B.-E. (2008). A systematic review of the success of sinus floor elevation and survival of implants inserted in combination with sinus floor elevation. Part II: Transalveolar technique. J. Clin. Periodontol..

[6] Rosen P.S., Summers R., Mellado J.R., Salkin L.-M., Shanaman R.-H., Marks M.-H., Fugazzotto P.A. (1999). The bone-added osteotome sinus floor elevation technique: Multicenter retrospective report of consecutively treated patients. Int. J. Oral. Maxillofac. Implants..

[7] Tatum H. (1986). Maxillary and sinus implant reconstructions. Dent. Clin. N. Am..

[8] Beretta M., Poli P.-P., Grossi G.-B., Pieroni S., Maiorana C. (2015). Long-term survival rate of implants placed in conjunction with 246 sinus floor elevation procedures: Results of a 15-year retrospective study. J. Dent..

[9] Farina R., Franceschetti G., Travaglini D., Consolo U., Minenna L., Schincaglia G.P., Trombelli L., Riccardi O., Bandieri A., Maietti E. (2019). Radiographic outcomes of transcrestal and lateral sinus floor elevation: One-year results of a bi-center, parallel-arm randomized trial. Clin. Oral. Impl. Res..

[10] Romero-Millán J., Martorell-Calatayud L., Peñarrocha M., García-Mira B. (2012). Indirect osteotome maxillary sinus floor elevation: An update. J. Oral. Implantol..

[11] Maximiano Millán A., Bravo Álvarez R., Plana Montori M., Guerrero González M., Saura García-Martín D., Ríos-Carrasco B., Fernández-Palacín A., Monticelli F., Ríos-Santos J.V., Fernández-Palacín A. (2020). Assessment of the simultaneous use of biomaterials in transalveolar sinus floor elevation: Prospective randomized clinical trial in humans. Int. J. Environ. Res. Public Health.

[12] Yin L., Yu Z., Chen Z., Huang B., Zhang K., Zhou A., Li X. (2016). Analysis of bone height changes after maxillary sinus augmentation with simultaneous and delayed placement of dental implants: A clinical and radiographic study. J. Prosthodont..

[13] Correia F., Pozza D.H., Gouveia S., Felino A.C., Faria-Almeida R. (2021). Advantages of porcine xenograft over autograft in sinus lift: A randomised clinical trial. Materials.

[14] Velasco-Ortega E., Valente N.-A., Iezzi G., Petrini M., Derchi G., Barone A., Valente N.-A., Iezzi G., Petrini M., Derchi G. (2021). Maxillary sinus augmentation with three different biomaterials: Histologic, histomorphometric, clinical and patient reported outcomes from a randomized controlled trial. Clin. Impl. Dent. Relat. Res..

[15] Nasr S., Slot D.-E., Bahaa S., Dorfer C.-E., Fawzy El-Sayed K.-M. (2016). Dental implants combined with sinus augmentation: What is the merit of bone grafting? A systematic review. J. Cranio Maxillofac. Surg..

[16] Alayan J., Ivanovski S. (2018). A prospective controlled trial comparing xenograft/autogenous bone and collagen-stabilized xenograft for maxillary sinus augmentation—Complications, patient-reported outcomes and volumetric analysis. Clin. Oral. Impl. Res..

[17] Eliaz N., Metoki N. (2017). Calcium Phosphate Bioceramics: A Review of Their History, Structure, Properties, Coating Technologies and Biomedical Applications. Materials.

[18] Da Silva Brum I., Frigo L., Goncalo Pinto Dos Santos P., Nelson Elias C., da Fonseca G.A.M.D., Jose de Carvalho J. (2021). Performance of Nano-Hydroxyapatite/Beta-Tricalcium Phosphate and Xenogenic Hydroxyapatite on Bone Regeneration in Rat Calvarial Defects: Histomorphometric, Immunohistochemical and Ultrastructural Analysis. Int. J. Nanomed..

[19] Badwelan M., Alkindi M., Alghamdi O., Ahmed A., Ramalingam S., Alrahlah A. (2021). Bone Regeneration Using PEVAV/β-Tricalcium Phosphate Composite Scaffolds in Standardized Calvarial Defects: Micro-Computed Tomographic Experiment in Rats. Materials.

[20] López-López J., Chimenos-Küstner E., Manzanares-Cespedes C., Muñoz-Sánchez J., Castañeda-Vega P., Jané-Salas E., Álvarez López J.M., Gimeno Sandig A. (2009). Histomorphological study of the bone regeneration capacity of platelet-rich plasma, bone marrow and tricalcium phosphate: Experimental study on pigs. Med. Oral. Patol. Oral. Cir. Bucal..

[21] Cirera A., Manzanares M.-C., Sevilla P., Ortiz-Hernandez M., Galindo-Moreno P., Gil J. (2019). Biofunctionalization with a TGFβ-1 Inhibitor Peptide in the Osseointegration of Synthetic Bone Grafts: An in Vivo Study in Beagle Dogs. Materials.

[22] Shalash M.-A., Rahman H.-A., Azim A.-A., Neemat A.-H., Hawary H.-E., Nasry S.-A. (2013). Evaluation of horizontal ridge augmentation using beta tricalcium phosphate and demineralized bone matrix: A comparative study. J. Clin. Exp. Dent..

[23] Wu J., Jiang J.-H., Xu L., Liang C., Bai Y., Zou W. (2015). Apilot clinical study of Class III surgical patients facilitated by improved accelerated osteogenic orthodontic treatments. Angl. Orthod..

[24] Choy C.-S., Lee W.-F., Lin P.-Y., Wu Y.-F., Huang H.-M., Teng N.C., Pan Y.H., Salamanca E., Chang W.J. (2021). Surface Modified β-Tricalcium phosphate enhanced stem cell osteogenic differentiation in vitro and bone regeneration in vivo. Sci. Rep..

[25] Cömert Kılıç S., Güngörmüs M., Parlak S.-N. (2017). Histologic and histomorphometric assessment of sinus-floor augmentation with beta-tricalcium phosphate alone or in combination with pure-platelet-rich plasma or platelet-rich fibrin: A randomized clinical trial. Clin. Implant. Dent. Relat. Res..

[26] Kolerman R., Nissan J., Rahmanov M., Vered H., Cohen O., Tal H. (2017). Comparison between mineralized cancellous bone allograft and an alloplast material for sinus augmentation: A split mouth histomorphometric study. Clin. Implant. Dent. Relat. Res..

[27] Al-Moraissi E.-A., Alkhutari A.-S., Abotaleb B., Altairi N.-H., Del Fabbro M. (2020). Do osteoconductive bone substitutes result in similar bone regeneration for maxillary sinus augmentation when compared to osteogenic and osteoinductive bone grafts? A systematic review and frequentist network meta-analysis. Int. J. Oral. Maxillofac. Surg..

[28] Aragoneses-Lamas J.M., Gómez-Sánchez M., Cuadrado-González L., Suárez-García A., Aragoneses Sánchez J. (2020). Vertical bone gain after sinus lift procedures with beta-tricalcium phosphate and simultaneous implant placement—a cross-sectional study. Medicina.

[29] Gorla L.-F., Spin-Neto R., Boos F.-B., Pereira Rdos S., Garcia-Junior I.-R., Hochuli-Vieira E. (2015). Use of autogenous bone and beta-tricalcium phosphate in maxillary sinus lifting: A prospective, randomized, volumetric computed tomography study. Int. J. Oral. Maxillofac. Surg..

[30] La Monaca G., Iezzi G., Cristalli M.-P., Pranno N., Sfasciotti G.-L., Vozza I. (2018). Comparative histological and histomorphometric results of six biomaterials used in two-stage maxillary sinus augmentation model after 6-month healing. Biomed. Res. Int..

[31] Loin J., Kün-Darbois J.D., Guillaume B. (2019). Badja, S.; Libouban, H.; Chappard, D. Maxillary sinus floor elevation using Beta-Tricalcium-Phosphate (beta-TCP) or natural bone: Same inflammatory response. J. Mater. Sci. Mater. Med..

[32] Park W.-B., Han J.-Y., Kang K.-L. (2021). Long-term comparison of survival and marginal bone of implants with and without sinus augmentation in maxillary molars within the same patients: A 5.8- to 22-year retrospective study. J. Clin. Med..

[33] Szabó G., Huys L., Coulthard P., Maiorana C., Garagiola U., Barabás J., Németh Z., Hrabák K., Suba Z. (2005). A prospective multicenter randomized clinical trial of autogenous bone versus beta-tricalcium phosphate graft alone for bilateral sinus elevation: Histologic and histomorphometric evaluation. Int. J. Oral. Maxillofac. Implant..

[34] Corbella S., Taschieri S., Weinstein R., del Fabbro M. (2016). Histomorphometric outcomes after lateral sinus floor elevation procedure: A systematic review of the literature and meta-analysis. Clin. Oral. Implant. Res..

[35] Velich N., Németh Z., Tóth C., Szabó G. (2004). Long-term results with different bone substitutes used for sinus floor elevation. J. Craniofac. Surg..

[36] Zijderveld S.A., Schulten E.A.J.M., Aartman I.H.A., ten Bruggenkate C.M. (2009). Long-term changes in graft height after maxillary sinus floor elevation with different grafting materials: Radiographic evaluation with a minimum follow-up of 4.5 years. Clin. Oral. Impl. Res..

[37] Okada T., Kanai T., Tachikawa N., Munakata M., Kasugai S. (2016). Long-term radiographic assessment of maxillary sinus floor augmentation using beta-tricalcium phosphate: Analysis by cone-beam computed tomography. Int. J. Impl. Dent..

[38] Park W.-B., Kang K.L., Han J.-Y. (2019). Factors influencing long-term survival rates of implants placed simultaneously with lateral maxillary sinus floor augmentation: A 6- to 20-year retrospective study. Clin. Oral. Impl. Res..

[39] Chao Y.L., Chen H.H., Mei C.C., Tu Y.K., Lu H.K. (2010). Meta-regression analysis of the initial bone height for predict- ing implant survival rates of two sinus elevation procedures. J. Clin. Periodontol..

[40] Mordenfeld A., Albrektsson T., Hallman M. (2014). A 10-year clinical and radiographic study of implants placed after maxillary sinus floor augmentation with an 80:20 mixture of deproteinized bovine bone and autogenous bone. Clin. Implant. Dent. Relat. Res..

[41] Starch-Jensen T., Mordenfeld A., Becktor J.P., Jensen S.S. (2018). Maxillary sinus floor augmentation with synthetic bone substitutes compared with other grafting materials: A systematic review and meta-analysis. Impl. Dent..

[42] Díaz-Olivares L.A., Cortés-Bretón Brinkmann J., Martínez-Rodríguez N., Martínez-González J.M., López-Quiles J., Leco-Berrocal I., Meniz-García C. (2021). Management of Schneiderian membrane perforations during maxillary sinus floor augmentation with lateral approach in relation to subsequent implant survival rates: A systematic review and meta-analysis. Int. J. Impl. Dent..

[43] Rengo C., Fiorino A., Cucchi A., Nappo A., Randellini E., Calamai P., Ferrari M. (2021). Patient-reported outcomes and complication rates after lateral maxillary sinus floor elevation: A prospective study. Clin. Oral. Investig..

[44] Alayan J., Ivanovski S. (2019). Biological and technical outcomes of restored implants after maxillary sinus augmentation—Results at 1-year loading. Clin. Oral. Impl. Res..

[45] Stacchi C., Troiano G., Rapani A., Lombardi T., Sentineri R., Speroni S., Bertron F., Di Lenarda R. (2020). Factors influencing the prevalence of peri-implantitis in implants inserted in augmented maxillary sinuses: A multicenter cross-sectional study. J. Periodontol..

[46] Saaby M., Karring E., Schou S., Isidor F. (2016). Factors influencing severity of peri-implantitis. Clin. Oral. Impl. Res..

